# Supplementary material for: ProteinShader: illustrative rendering of macromolecules
Source: BMC Struct Biol. 2009 Mar 30;9:19. doi: 10.1186/1472-6807-9-19 (PMC2672931; doi:10.1186/1472-6807-9-19)
Supplement: Additional file 1 — ProteinShader program without source code. This compressed file contains the complete ProteinShader program including associated libraries, but no source code. A README.txt file gives an overview of the ProteinShader distribution, and the index.html file in the help subdirectory has directions on getting started with the program as well as a set of tutorials. [file 1472-6807-9-19-S1.zip › ProteinShader-beta-0_9_4-binary/help/api/org/proteinshader/gui/enums/class-use/JitterEnum.html]

Uses of Class org.proteinshader.gui.enums.JitterEnum (ProteinShader API)


|  |  |  |
| --- | --- | --- |
| **Overview** \| **Package** \| **Class** \| **Use** \| **Tree** \| **Deprecated** \| **Index** \| **Help** | |  |
| PREV   NEXT | **FRAMES**    **NO FRAMES**     **All Classes** |  |


---


## **Uses of Class org.proteinshader.gui.enums.JitterEnum**

| Packages that use JitterEnum | |
| --- | --- |
| **org.proteinshader.gui** | Holds all of the Swing GUI components and their associated listeners, including class Renderer, which is registered as a listener for the GLCanvas object that is used a drawing surface. |
| **org.proteinshader.gui.components.controlpanel** | Holds the JPanels used in the right-side retractable control panel of the GUI. |
| **org.proteinshader.gui.enums** | Holds the enums needed by org.proteinshader.gui and its subpackages. |

Uses of JitterEnum in org.proteinshader.gui

| Methods in org.proteinshader.gui with parameters of type JitterEnum | |
| --- | --- |
| `void` | `MediatorImpl.jitterScene(JitterEnum jitter)`             Draws the scene multiple times to an accumulation buffer while jittering the xy-coordinates a fraction of a pixel. |
| `void` | `Renderer.jitterScene(JitterEnum jitter)`             Draws the scene multiple times to an accumulation buffer while jittering the xy-coordinates a fraction of a pixel. |
| `void` | `Mediator.jitterScene(JitterEnum jitter)`             Draws the scene multiple times to an accumulation buffer while jittering the xy-coordinates a fraction of a pixel. |

Uses of JitterEnum in org.proteinshader.gui.components.controlpanel

| Fields in org.proteinshader.gui.components.controlpanel declared as JitterEnum | |
| --- | --- |
| `static JitterEnum` | `AntialiasingPanel.DEFAULT_JITTER`             Sets the initial menu selection for the number of times to jitter when applying antialiasing to the entire scene (n = 3). |

| Methods in org.proteinshader.gui.components.controlpanel with parameters of type JitterEnum | |
| --- | --- |
| `void` | `AntialiasingPanel.antialiasScene(JitterEnum jitter)`             The jitter value given as an argument is only applied to the scene if the 'Antialias entire scene' checkbox is currently selected. |

Uses of JitterEnum in org.proteinshader.gui.enums

| Methods in org.proteinshader.gui.enums that return JitterEnum | |
| --- | --- |
| `static JitterEnum` | `JitterEnum.valueOf(String name)`             Returns the enum constant of this type with the specified name. |
| `static JitterEnum[]` | `JitterEnum.values()`             Returns an array containing the constants of this enum type, in the order they're declared. |

---


|  |  |  |
| --- | --- | --- |
| **Overview** \| **Package** \| **Class** \| **Use** \| **Tree** \| **Deprecated** \| **Index** \| **Help** | |  |
| PREV   NEXT | **FRAMES**    **NO FRAMES**     **All Classes** |  |


---

# *Copyright © 2007-2008*
